# Health‐related quality of life in long‐term prostate cancer survivors after nerve‐sparing and non‐nerve‐sparing radical prostatectomy—Results from the multiregional PROCAS study

**DOI:** 10.1002/cam4.3197

**Published:** 2020-06-10

**Authors:** Salome Adam, Eva Martin‐Diener, Bertrand Camey, Céline Egger Hayoz, Isabelle Konzelmann, Seyed Mohsen Mousavi, Christian Herrmann, Sabine Rohrmann, Miriam Wanner, Katharina Staehelin, Räto T. Strebel, Marco Randazzo, Hubert John, Hans‐Peter Schmid, Volker Arndt

**Affiliations:** ^1^ National Institute for Cancer Epidemiology and Registration (NICER) c/o University of Zurich Zurich Switzerland; ^2^ Division of Chronic Disease Epidemiology, Epidemiology, Biostatistics and Prevention Institute University of Zurich Zurich Switzerland; ^3^ Fribourg Cancer Registry Fribourg Switzerland; ^4^ Health Observatory Valais Valais Cancer Registry Sion Switzerland; ^5^ Cancer Registry East Switzerland St. Gallen Switzerland; ^6^ Cancer Registry Graubünden and Glarus Chur Switzerland; ^7^ Cancer Registry Zurich Zug, Schaffhausen and Schwyz University Hospital Zurich Zurich Switzerland; ^8^ Basel Cancer Registry Cantonal Department of Health Basel Switzerland; ^9^ Department of Urology Graubünden Cantonal Hospital Chur Switzerland; ^10^ Department of Urology Winterthur Cantonal Hospital Winterthur Switzerland; ^11^ Department of Urology St. Gallen Cantonal Hospital St. Gallen Switzerland; ^12^ Unit of Cancer Survivorship Division of Clinical Epidemiology and Aging Research German Cancer Research Center (DKFZ) Heidelberg Germany

**Keywords:** health‐related quality of life, long‐term survivor, nerve‐sparing radical prostatectomy, prostate cancer, sexual outcomes, urinary outcomes

## Abstract

**Background:**

Nerve‐sparing (NS) surgery was developed to improve postoperative sexual and potentially urological outcomes after radical prostatectomy (RP). However, it is largely unknown how NSRP affects health‐related quality of life (HRQoL) including urinary and sexual outcomes in prostate cancer (PC) survivors 5‐10 years after diagnosis in comparison with Non‐NSRP.

**Methods:**

The study population included 382 stage pT2‐T3N0M0 PC survivors 5‐10 years post diagnosis, who were identified from the multiregional *Prostate Cancer Survivorship in Switzerland* (PROCAS) study. Briefly, in 2017/2018, PC survivors were identified via six population‐based cancer registries based in both German‐ and French‐speaking Switzerland. HRQoL and PC‐specific symptom burden was assessed using the EORTC QLQ‐C30 and EORTC QLQ‐PR25 questionnaires. Differences in HRQoL outcomes between survivors treated with NSRP (uni‐ & bilateral) and Non‐NSRP were analyzed with multivariable linear regression adjusted for age, years since diagnosis, cancer stage, comorbidities at diagnosis, and further therapies, if appropriate. Multiple imputation was performed to minimize the bias due to missing data.

**Results:**

Five to ten years after diagnosis, PC survivors treated with NSRP and Non‐NSRP reported similar symptom burden and comparable HRQoL function scores. The only significant differences were reported for sexual activity, whereas PC survivors who underwent NSRP reported statistically significant (*P* = .031) higher sexual activity than those on Non‐NSRP. NSRP and Non‐NSRP reported similar scores for urinary symptoms and all other HRQoL outcomes.

**Conclusions:**

Our results support nerve‐sparing techniques as an option to improve postoperative sexual, but not urinary outcomes after RP in long‐term PC survivors.

## Graphical abstract

Nerve‐sparing radical prostatectomy and non‐nerve‐sparing radical prostatectomy were generally associated with comparable long‐term health‐related quality of life outcomes, but nerve‐sparing radical prostatectomy was linked with significantly higher sexual activity scores than non‐nerve‐sparing radical prostatectomy. Our results support nerve‐sparing techniques as an option to improve postoperative sexual but not urinary outcomes after radical prostatectomy in long‐term prostate cancer survivors.

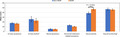

## 1 INTRODUCTION

Prostate Cancer (PC) remains the most frequently diagnosed cancer in men in many Western countries with an age‐adjusted cumulative incidence rate in Switzerland of 129.6/100,000.^1^ Over the last decades, PC prognosis has substantially improved resulting in a 5‐year relative survival rate of 90% in Switzerland.^2^ Although radical prostatectomy (RP) is the most common treatment strategy for men diagnosed with PC ^3^ with 88.2 RP/100’000 men in Switzerland,^1^ the survival benefit for PC survivors treated with RP compared to other primary management options remains unclear.^4^, ^5^, ^6^ For example, an analysis of PC survivors in the Swiss canton of Zurich revealed similar relative 1‐, 5‐, and 10‐year survival rates for PC survivors treated either with RP or radiotherapy.^7^ In addition, RP may have both strong acute and long‐lasting detrimental effects, notably on urinary and sexual functioning.^8^, ^9^, ^10^, ^11^

Nerve‐sparing (NS) technique was developed to improve postoperative sexual function (SEF) ^12^ and potentially urinary outcomes after RP. Indeed, higher recovery rates of SEF after nerve‐sparing RP compared to non‐nerve‐sparing (Non‐NS) RP could be observed up to 24 months.^13^, ^14^ However, a positive effect for postoperative urinary outcomes remains controversial.^15^, ^16^ For example, a study performed at the cantonal hospital Graubünden among 453 PC patients treated with robot‐assisted RP, whereas 265 patients (58.5%) were operated nerve‐sparing, showed that planed nerve‐sparing was not associated with an improved continence rate.^17^ A systematic review published in 2015 concluded that preservation of the neurovascular bundles improves urinary continence in the first 6 months after surgery, but no effect was seen for later time points up to 5 years after treatment.^18^

Even though extensive research was performed regarding whether NSRP improves urinary outcomes and SEF compared to Non‐NSRP in the first time 6‐24 months after the operation, little attention has been given to long‐term effects. Also research regarding whether NSRP compared to Non‐NSRP affects general health‐related quality of life (HRQoL) remains limited. To the authors’ knowledge, only one study from the United States ^19^ has assessed HRQoL domains (physical functioning and mental health) and disease‐specific symptom burden in PC survivors up to 10 years after diagnosis either treated with NSRP or Non‐NSRP. In this longitudinal study, PC survivors treated with Non‐NSRP reported similar declines at 5 years after diagnosis in physical functioning, mental health, and urinary continence scores compared to survivors treated with NSRP but significantly stronger declines in SEF scores. However, no study assessed further HRQoL functions and cancer and treatment‐related symptom burden beside urinary outcomes and SEF in long‐term PC survivors. Therefore, the objective of our study was to identify differences and similarities in general HRQoL and PC‐specific symptom burden by NSRP (uni/bilateral) and Non‐NSRP in long‐term PC survivors.

## 2 MATERIAL AND METHODS

### 2.1 Study design and study population

**Figure 1:**
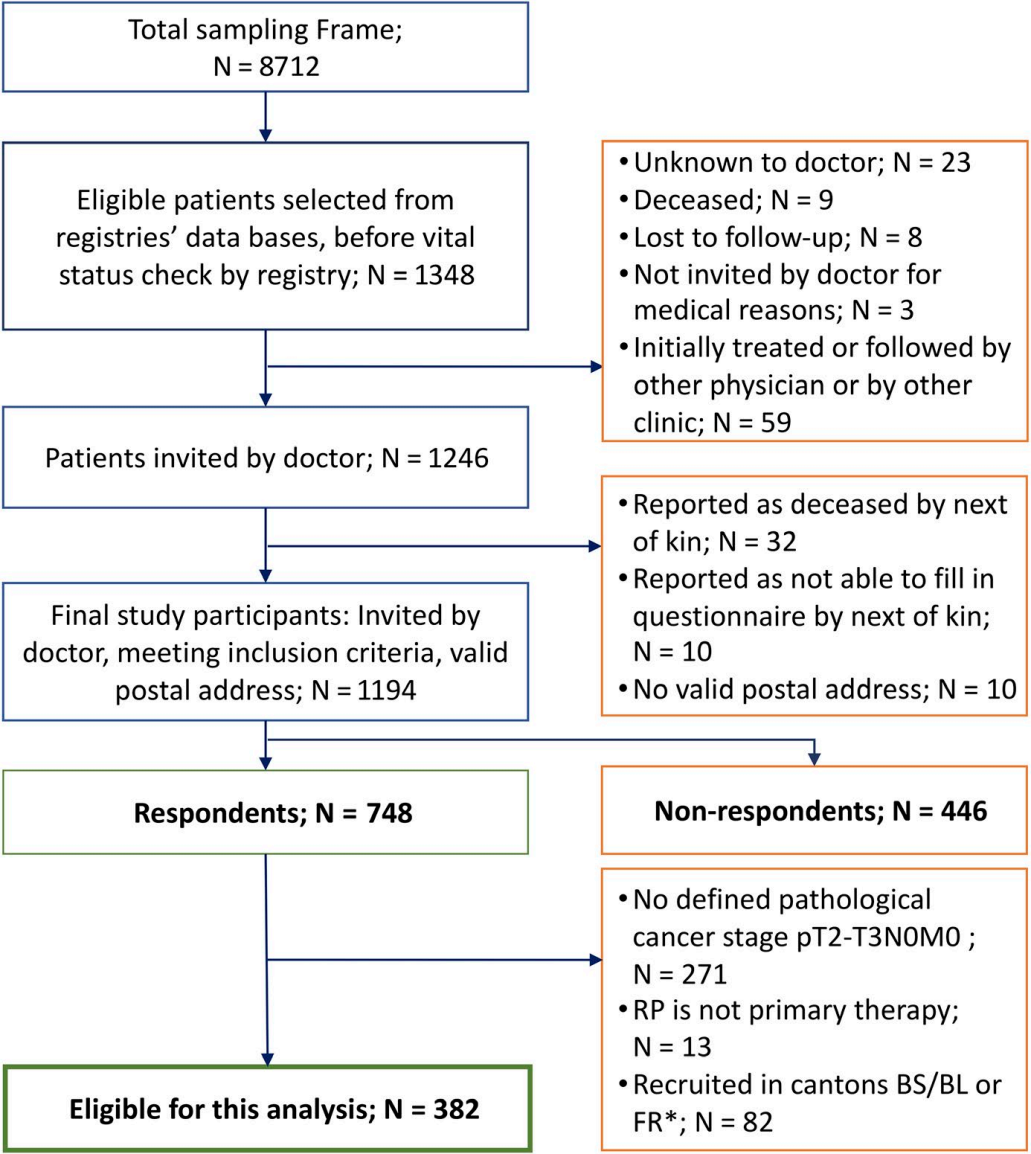
Study Flowchart. *For PC survivors in the cantons of Basel‐Stadt (BS), Basel‐Landschaft (BL), and Fribourg (FR) information on degree of NSRP was not available

Participants were included from the multiregional *Prostate Cancer Survivorship in Switzerland* (PROCAS) cohort. Details of the PROCAS study recruitment and data collection design have been described elsewhere.^20^ In short, the PROCAS study included 748 long‐term PC survivors who were diagnosed between 2006 and 2011 and aged 42‐75 years at diagnosis. They were identified via six population‐based cancer registries (Cancer Registry Fribourg, Cancer Registry Basel, Cancer Registry Graubünden and Glarus, Cancer Registry East Switzerland, Valais Cancer Registry & Cancer Registry Zurich and Zug) covering an underlying population of 3 456 020 million inhabitants, based in both German and French‐speaking Switzerland and invited via their referring urologists. Data collection was conducted between 2017 and 2018 by postal questionnaire. Nonrespondents received one reminder. Among PC survivors, 8712 fulfilled the inclusion criteria for the study (Figure 1). Of them, 1246 PC survivors were randomly selected for participation. 1194 could be contacted and received an invitation. Finally, 748 returned a completed questionnaire (response rate: 62.2%). This analysis was restricted to PC survivors (a) staged pT2‐T3 N0 and M0 (according to the TNM classification system published by the American Joint Committee on Cancer have been available in the FCD since 2002^21^), (b) treated with RP as primary therapy, and (c) recruited from cancer registries (Cancer Registry Graubünden and Glarus, Cancer Registry East Switzerland, Valais Cancer Registry & Cancer Registry Zurich and Zug), which provided information on degree of NSRP (N = 382). Our sample was restricted to pT2‐T3N0M0 cases to create a homogenous cohort, because PC patients staged pT4 or with cancer in lymph nodes or metastases need different treatment strategies.

## 3 STUDY MEASURES

### 3.1 HRQoL and PC‐specific symptom burden

HRQoL and PC‐specific symptom burden were assessed once (cross‐sectional design) with internationally validated instruments: the EORTC QLQ‐C30 and the PC‐specific module QLQ‐PR25. The EORTC QLQ‐C30 questionnaire consists of 30 items, comprising five functioning scales, a global health/quality of life (QoL) scale, and nine items/scales on symptoms and financial difficulties. The PC‐specific EORTC QLQ‐PR25 questionnaire consists of 25 questions, assessing urinary and bowel symptoms, sexual activity, sexual functioning, and hormonal treatment‐related symptoms. Scoring of all instruments was performed according to the pertinent scoring manuals.^22^, ^23^ High scores on the functioning scales and global health/QoL indicate better functioning and better health, respectively. For the symptom and financial difficulty scales, a higher score represents a greater symptom burden and financial difficulty. High scores in the EORTC QLQ‐PR25 represent a greater symptom burden or a better sexual functioning and more sexual activity.

### 3.2 Demographics, lifestyle, and clinical data

Information whether patients were treated with NSRP and whether it was uni‐ or bilateral NSRP were provided by the treating urologist, together with the cantonal and regional cancer registries. Assessment whether NSRP was performed nerve‐sparing was based on the surgeons’ appraisal. Physicians also gave detailed information on other treatments, disease progression/ relapse (including biochemical and clinical recurrence and metastasis after diagnosis of primary tumor at time of survey) and other primary tumors. Cancer registries provided demographic parameters and clinical information such as date of birth, date of diagnosis and cancer stage. Self‐reported demographic included education, living with partner, working status, weight, height, and nationality. Questionnaires and all other study documents were available in German, French, and Italian.

## 4 STATISTICS

For descriptive purposes, we compared Non‐NSRP (n = 167) vs NSRP (n = 215) by several clinical and sociodemographic characteristics. The group NSRP consisted of PC survivors treated with both uni‐ and bilateral NSRP, as these two groups did not differ with respect to clinical and sociodemographic characteristics (Table S1) and reported similar HRQoL outcomes (Tables S2). Additionally, we compared uni‐ and bilateral NSRP with Non‐NSRP separately in order to determine whether outcomes of unilateral NSRP and Non‐NSRP might be more similar to each another than they were to bilateral NSRP.

Adjusted means based on multivariable linear regression models were calculated to describe and test for differences in HRQoL. Models were adjusted for age at survey, years since diagnosis, cancer stage at diagnosis, comorbidities at diagnosis, and further therapy (during 1st year after diagnosis) if appropriate. Other variables including education, working status, nationality, and language were considered as additional potential confounders, but not included as they did not improve the model fit.

To assess the effect of age and disease progression/relapse, we performed sensitivity analysis: (a) excluding all patients with a disease progressions/relapse and (b) stratifying by age using the mean of 72.4 years as cut‐off.

Multiple Imputation Chained Equations (MICE) procedure with 25 repetitions was used to reduce possible bias due to missing values. MICE is the standard procedure in epidemiological research under the missing‐at‐random assumption.^24^ It has the advantage that missing values are filled multiple times “(…) based on the observed values for a given individual and the relation observed in the data for other participants (…)”.^25^ Therefore, the analyses of the multiple imputed data consider the uncertainty in the imputed data, resulting in accurate standard errors.^25^ Differences in mean HRQoL scores larger than 10 points were considered clinically meaningful.^26^ A *P*‐value < .05 (two‐sided) was considered statistically significant. The *P*‐values were not adjusted for multiple testing, so the *P*‐values refer to the individual tests rather than a global test for differences. All analyses were performed using STATA statistical software (Version 15.1).

## 5 RESULTS

Overall, from 382 PC survivors included in this analysis 56.3%, received NSRP and 43.7% Non‐NSRP (Table 1). Mean age was 72.4 years and mean time since diagnosis was 7.5 years. Participants had mainly Swiss nationality and filled‐in the German questionnaire. Beside years since diagnosis (*P* = .026) and disease progression (*P* = .029), clinical and demographic characteristics were comparable among PC survivors by treatment strategy. For PC survivors treated with Non‐NSRP mean time since diagnosis was 7.8 years, whereas it was 7.4 years for PC survivors treated with NSRP. Moreover, disease progression/relapse was more likely among participants on Non‐NSRP (28.8% vs 17.7%).

**Table 1:** Demographic and clinical characteristics of PC survivors by nerve‐sparing status (after multiple imputation of missing values)

	Total	Non‐NSRP	NSRP	Non‐NSRP vs NSRP
	(n = 382)	(n = 167)	(n = 215)	*P*‐value
	Col%	Col%	Col%	
Age at survey				
<70 years	30.4	29.6	31.7	
70‐74 years	32.2	31.1	33.0	
75‐79 years	25.1	25.0	25.2	
≥80 years	12.3	15.2	10.1	
Mean (SD)	72.4 (6.3)	72.9 (6.5)	71.9 (6.3)	.236
Education (highest degree)^a^				
Low	0.8	0.6	0.9	
Medium	51.2	50.3	51.8	
High	48.0	49.1	47.3	.572
Nationality Swiss (yes)	95.5	96.0	95.2	.752
Language questionnaire				
German	94.8	97.0	93.0	
French/Italian	5.2	3.0	7.0	.127
Living with partner (yes)	82.3	78.1	85.5	.197
Working at survey (yes)	11.8	10.4	12.9	.54
Body Mass Index				
<18.5	0.3	0.6	0.0	
18.5‐24.9	35.1	31.1	38.0	
25.0‐29.9	52.0	51.6	51.6	
≥30	12.6	16.8	10.4	.212
Cancer stage				
pT2N0M0	77.4	72.8	82.0	
pT3N0M0	22.6	27.2	18.0	.087
Years since diagnosis				
5‐6	27.2	22.3	31.0	
7‐8	46.1	43.8	47.8	
9‐10	26.7	33.9	21.1	
Mean (SD)	7.5 (1.4)	7.8 (1.5)	7.4 (1.5)	.026
Disease progression/relapse (yes)	22.0	28.4	17.7	.029
Comorbidities at diagnosis				
0	72.4	80.0	66.9	
1	19.1	12.2	24.2	
≥2	8.5	7.8	8.9	.091
Further therapy (during 1st year after diagnosis)				
External‐beam radiation therapy	6.6	7.8	5.6	.584
Hormone therapy	4.0	4.3	3.7	.850

Abbreviation: Col, Column.

^a^ Education: Low (no or primary school); Medium (lower general secondary education or vocational training); High (pre‐university education, high vocational training, university).

Multiple imputation did not substantially alter the distribution of Non‐NSRP and NSRP (Table S3) and their association with baseline clinical and demographic characteristics (Tables S4) and HRQoL outcomes (Table S5).

### 5.1 HRQoL and PC‐specific symptom burden of PC survivors treated with Non‐NSRP vs NSRP

**Figure 2:**
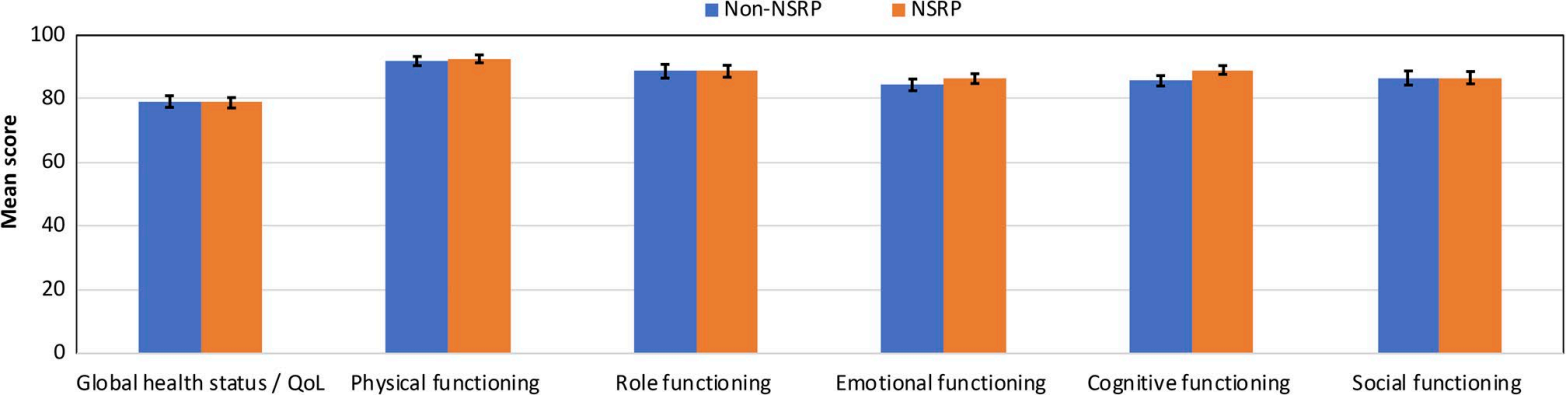
Adjusted mean scores of EORTC QLQ‐C30 HRQoL scales of PC survivors by nerve‐sparing status (after multiple imputation of missing values). A high score represents a high/healthy level of functioning/high QoL. Mean scores were adjusted for age at survey, years since diagnosis, cancer stage, comorbidities at diagnosis, and further therapy if appropriate. I bars represent ± standard errors; all *P*‐values > .05

In general, PC survivors reported excellent functioning and good general health/overall quality of life. Adjusted mean scores of all functioning scales were comparable between PC survivors who have received Non‐NSRP and NSRP (Figure 2, all *P*‐values > .05).

**Figure 3:**
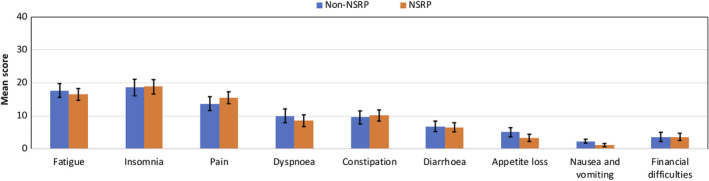
Adjusted mean of EORTC QLQ‐C30 symptom scales of PC survivors by nerve‐sparing status (after multiple imputation of missing values). A high score represents a high symptom burden. Mean scores were adjusted for age at survey, years since diagnosis, cancer stage, comorbidities at diagnosis, and further therapy if appropriate. I bars represent ± standard errors; all *P*‐values > .05

Fatigue, insomnia and pain were the symptoms with the highest reported burden among all PC survivors. However, differences in adjusted mean scores between patients treated with Non‐NSRP and NSRP were small (max. 2.8 scale points) and not statistically significant (Figure 3, all *P*‐values > .05).

**Figure 4:**
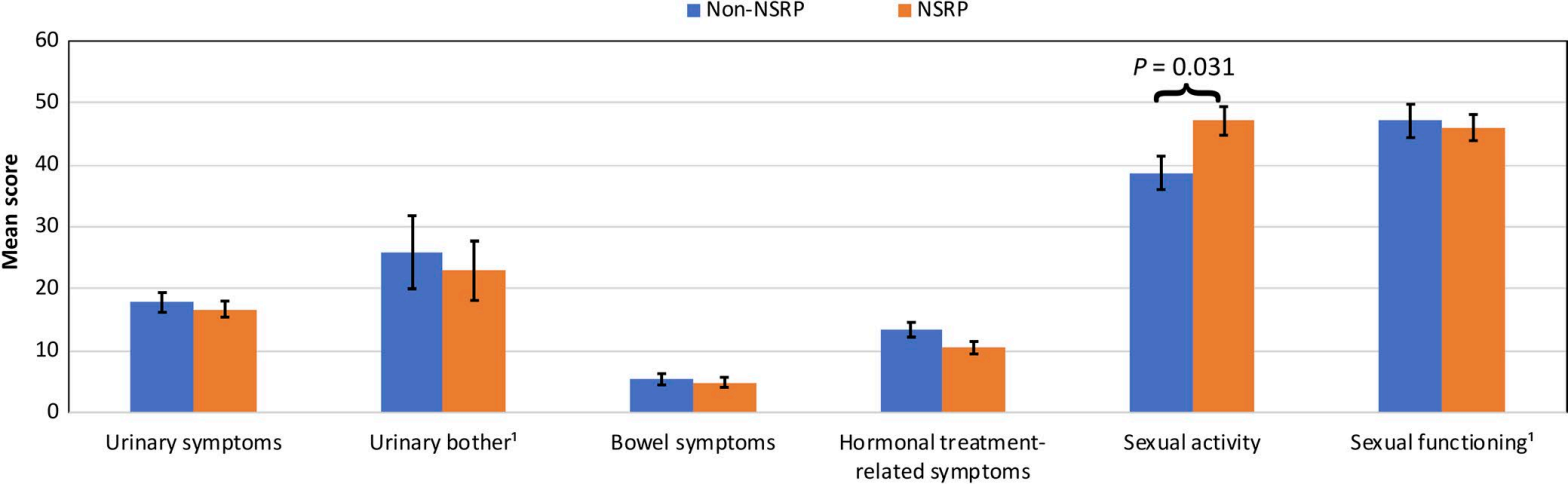
Adjusted mean of EORTC PR25 scales of PC survivors by nerve‐sparing status (after multiple imputation of missing values).A high score represents higher symptom burden or higher sexual activity/ better sexual functioning. Mean scores were adjusted for age at survey, years since diagnosis, cancer stage, comorbidities at diagnosis and further therapy if appropriate.^1^ smaller sample size as questions regarding these functions were conditional—urinary bother (n = 104) & sexual functioning (n = 183)I bars represent ± standard errors; all *P*‐values > .05 if not indicated otherwise

PC survivors who underwent NSRP reported statistically significant (*P* = .031) higher sexual activity (not clinically significant different), but similar sexual functioning compared to those who have received Non‐NSRP (Figure 4). Regarding urinary symptoms and urinary bother no significant different mean scores were reported.

Neither differences in urinary symptoms (n = 132), urinary bother (n = 37) nor sexual activity (n = 131) and sexual function (n = 73) were observed when comparing HRQoL and PC‐specific symptom burden of PC survivors treated with unilateral NSRP vs bilateral NSRP (Table S2). When unilateral and bilateral NSRP were compared to Non‐NSRP separately, results showed a similar pattern, indicating that unilateral NSRP outcomes were not more similar to Non‐NSRP than to bilateral NSRP (data not shown).

Restriction to long‐term PC survivors without disease progression/relapse did not substantially alter the pattern of our findings (data not shown). Furthermore, the pattern of our findings was similar in age‐stratified analyses. However, younger long‐term survivors (<72.4 years of age) reported higher HRQoL functioning scores and lower symptom burden scores (Table S6 & Table S7). Even though younger long‐term PC survivors treated with NSRP reported a 6.3‐point higher score for sexual activity, this difference was statistically significant (*P* = .043) and clinically meaningful only in older long‐term PC survivors.

## 6 DISCUSSION

Given the increasing numbers of long‐term PC survivors,^27^ it is imperative to understand whether treatment modalities such as NSRP may result in long‐lasting health benefits including better HRQoL and lower PC‐specific symptom burden. This population‐based study suggests, however, that HRQoL and symptom burden in PC survivors 5‐10 years after diagnosis of localized PC in general does not vary according to type of surgery (NSRP versus Non‐NSRP) except for the finding that PC survivors who underwent NSRP were significantly more sexually active than those treated with Non‐NSRP.

In general, the results from our cross‐sectional survey confirm and extend the results of the longitudinal survey based on the CaPSURE registry.^19^ In addition to the results from the CaPSURE registry which described differences in SEF but comparable physical and mental health in PC survivors after NSRP and Non‐NSRP, there appears to be no further differences between the two treatment groups with respect to global health status, role, emotional, cognitive, and social functioning as well as burden of fatigue, insomnia, dyspnoea, constipation and pain according to our study. The result that no differences were observed in any non‐PC‐specific HRQoL and symptom burden domains is in line with previous studies, which reported little to no difference in HRQoL of long‐term PC survivors between treatment modalities ^9^ or in comparison with the general population.^11^ An explanation for this finding might be the response shift phenomenon, which results in a change in perception of burden threshold^28^, ^29^ after a cancer diagnosis. Even though long‐term PC survivors treated with Non‐NSRP suffer from worse SEF than survivors treated with NSRP, there is no impact on other HRQoL domains.

Previous studies have reported that NSRP improves postoperative sexual function up to five^13^, ^14^, ^30^ and 10 years after diagnosis^19^ compared to Non‐NSRP. In our study, no significant differences for SEF were reported, but patients treated with NSRP scored significantly higher on all sexual activity scales. The discrepancy between our results and the aforementioned studies is potentially based on the usage of different instruments to assess PC‐specific symptom burden. In this study, the EORTC‐PR25 questionnaire was used, whereas the other studies used the UCLA‐PCI and the EPIC‐26 questionnaire. In the EORTC‐PR25, sexual activity is measured with two questions, which are asking about sexual interest and frequency of sexual activity. Only patients who were sexually active (183/382) answered the additional questions on the ability to have an erection and orgasm, and sexual desire,^23^ which corresponds to the SEF domain. In contrast, all these items including level of sexual activity are part of the SEF domain in the other two instruments.^31^ Therefore, results regarding SEF from studies using different QoL instruments are not directly comparable. Nevertheless, our finding that NSRP results in favourable sexual function even 5‐10 years after diagnosis has potential clinical implications, even though the difference was small (not clinically meaningful), indicating that the impact may be less important for the individual. However, as the number of long‐term PC survivors is substantially growing,^32^ a large number of individuals are affected. Moreover, most PC survivors have very good survival perspectives and live with their partners. Therefore, we believe that our results might have an impact on the treatment decision‐making process.

In this context, the focus should be on older long‐term PC survivors. Even though, as indicated in an analysis of long‐term PC survivors in Germany,^11^ HRQoL scores are probably lower and symptom burden scores higher in older long‐term PC survivors; the differences in sexual activity in older PC survivors were not only statistically significant but also larger (clinically meaningful) than in younger survivors. Thus, the chances to preserve sexual activity should also play a role in elderly patient groups when deciding for a treatment strategy. However, these results need to be interpreted with caution due to the small sample size.

The lack of differences with respect to long‐term urinary symptoms and urinary bother might be surprising, but our results are similar to another study performed in Switzerland,^16^ even though studies differ methodologically. The latter study continuously assessed and compared urinary incontinence over a 10‐year period in PC survivors either treated with NSRP or Non‐NSRP. This study was conducted in a canton, which was not part of this study, and adjusted its models for factors (such as PC volume, clinical risk group, positive margins, and preoperative urinary incontinence score and PSA‐values), which were not assessed in our study. Beside those differences, the similarity of the results supports the conclusion of the other study, stating that NSRP should not be conducted with the primary aim to improve urinary outcomes.

To our knowledge, this is the first study performed in Europe which compared HRQoL and PC‐specific symptom burden according to NS surgery in long‐term PC survivors, using a multiregional‐based design, including patient recruitment via multiple population‐based cancer registries in two different language regions. Beside these strengths, there are limitations which need to be discussed. For example, not in all study regions, information was available whether participants received NSRP. Therefore, participants of two study regions were excluded from this analysis. Moreover, although we corrected our models for a range of clinical and sociodemographic variables, we did not have all information such as clinical risk group, the degree of nerve‐sparing, urinary outcome scores and most importantly baseline SEF, which might have influenced either the treatment choice or the outcome. Additionally, we were not able to identify trends over time, as HRQoL in the PROCAS study was assessed only once for each participant. Other studies indicated that HRQoL of PC survivors appears to be lower during and shortly after treatment, but to improve and stabilize thereafter.^8^, ^33^, ^34^ However, due to the cross‐sectional design of our study, causality between NSRP and better sexual outcomes cannot be established. Finally, information on whether RP was performed open or robotic‐assisted was not assessed in the study, what might also influence the generalizability of our results even though these two techniques yielded similar functional outcomes in short‐term PC survivors as seen in randomized controlled trials.^35^

## 7 CONCLUSION

In conclusion, NSRP was generally associated with comparable long‐term HRQoL outcomes but higher sexual activity scores when compared to Non‐NSRP. Our results support nerve‐sparing techniques as an option to improve post‐operative sexual but not urinary outcomes after RP in PC survivors.

## ETHICAL CONSENT

The PROCAS study has been approved as a multi‐centre study by the Ethics Committee Zurich and by all reviewer boards accountable for the participating cancer registries (BASEC Number: 2016‐00608). All procedures involving human participants were in accordance with the Helsinki Declaration of 1975, as revised in 1983.

## CONFLICT OF INTEREST

The authors declare that they have no competing interests.

## AUTHORS' CONTRIBUTIONS

SA & VA developed the study concept. SA, VA, EMD and HPS were responsible for the study design. Date acquisition was performed by SA, EMD, BC, CEH, IK, SMM; CH, SR, MQ, KS, RTS, MR, HJ, HPS & VA. SA performed the statistical analysis. SA and VA wrote the manuscript. All authors read, commented, and approved the final manuscript.

## Supporting information

Supplementary Material

## Data Availability

The data that support the findings of this study are available on request from the corresponding author. The data are not publicly available due to privacy or ethical restrictions.
