# Supplementary material for: Health‐related quality of life in long‐term prostate cancer survivors after nerve‐sparing and non‐nerve‐sparing radical prostatectomy—Results from the multiregional PROCAS study
Source: Cancer Med. 2020 Jun 10;9(15):5416–24. doi: 10.1002/cam4.3197 (PMC7402816; doi:10.1002/cam4.3197)
Supplement: Supplementary file 1 — Supplementary Material [file CAM4-9-5416-s001.docx]

**Supplementary Material**

| **Table S1:** **Demographic and clinical characteristics of PC survivors by nerve-sparing status** | | | |  |
| --- | --- | --- | --- | --- |
|  | **Unilateral NSRP** | **Bilateral NSRP** | **Uni- vs. Bilateral NSRP** | |
|  | (n=65) | (n=67) |  | |
|  | **Col%** | **Col%** | **p-value** | |
| **Age at survey** |  |  |  | |
| <70 years | 29.2 | 28.4 |  | |
| 70-74 years | 32.3 | 32.8 |  | |
| 75-79 years | 30.8 | 23.9 |  | |
| ≥80 years | 7.7 | 14.9 | 0.715 | |
| Mean (SD) | 72.4 (6.0) | 72.6 (6.3) | 0.844 | |
| **Education^1^ (highest degree)** |  |  |  | |
| Low | 1.5 | 0.0 |  | |
| Medium | 64.6 | 56.7 |  | |
| High | 33.9 | 43.3 | 0.228 | |
| **Nationality Swiss (yes)** | 96.7 | 95.5 | 0.673 | |
| **Language questionnaire** |  |  |  | |
| German | 90.8 | 86.6 |  | |
| French/Italian | 9.2 | 13.4 | 0.358 | |
| **Living with partner (yes)** | 87.0 | 80.3 | 0.334 | |
| **Working at survey (yes)** | 9.2 | 13.3 | 0.447 | |
| **Body-Mass-Index** |  |  |  | |
| <18.5 | 0.0 | 0.0 |  | |
| 18.5–24.9 | 27.7 | 41.8 |  | |
| 25.0–29.9 | 61.5 | 44.8 |  | |
| ≥30 | 10.8 | 7.5 |  | |
| Missing | 0.0 | 6.0 | 0.062 | |
| **Cancer stage** |  |  |  | |
| pT2N0M0 | 80.0 | 85.1 |  | |
| pT3N0M0 | 20.0 | 14.9 | 0.444 | |
| **Years since diagnosis** |  |  |  | |
| 5-6 | 38.5 | 23.9 |  | |
| 7-8 | 41.5 | 52.2 |  | |
| 9-10 | 20.0 | 23.9 | 0.132 | |
| Mean (SD) | 7.2 (1.5) | 7.4 (1.5) | 0.427 | |
| **Disease progression/relapse** | 23.1 | 11.9 | 0.092 | |
| **Comorbidities at diagnosis** |  |  |  | |
| 0 | 50.8 | 43.3 |  | |
| 1 | 16.9 | 16.4 |  | |
| 2 | 4.6 | 9.0 |  | |
| Missing | 27.7 | 31.3 | 0.121 | |
| **Further therapy (during 1^st^ year after diagnosis)** |  |  |  | |
| External-beam radiation therapy | 3.1 | 4.5 | 0.673 | |
| Hormone therapy | 3.1 | 0.0 | 0.148 | |
| Col. – Column  Information about NSRP status was complete.  ^1^ Education: Low (no or primary school); Medium (lower general secondary education or vocational training); High (pre-university education, high vocational training, university) | | | |  |

| **Table S2: Adjusted mean EORTC QLQ-C30 and EORTC PR-25 scores of PC survivors by nerve-sparing status** | | | | | | | |
| --- | --- | --- | --- | --- | --- | --- | --- |
|  | **Unilateral NSRP** | | **Bilateral NSRP** | | **Difference Unilateral – Bilateral NSRP** | | |
|  | **Mean** | **SE** | **Mean** | **SE** | **p-value** | **Mean** |  |
| **EORTC QLQ-C30 Functioning scales** |  |  |  |  |  |  |  |
| **Global health/QoL** | 78.1 | 2.2 | 77.1 | 2.2 | 0.713 | 1.0 |  |
| **Physical functioning** | 90.7 | 1.9 | 90.0 | 1.8 | 0.943 | 0.7 |  |
| **Cognitive functioning** | 89.1 | 2.8 | 90.7 | 2.8 | 0.645 | -1.6 |  |
| **Emotional functioning** | 86.0 | 2.2 | 84.8 | 2.2 | 0.658 | 1.2 |  |
| **Role functioning** | 86.0 | 2.2 | 84.8 | 2.2 | 0.205 | 1.2 |  |
| **Social functioning** | 84.6 | 3.0 | 85.4 | 3.0 | 0.831 | -0.8 |  |
| **EORTC QLQ-C30 Symptom scales** |  |  |  |  |  |  |  |
| **Fatigue** | 16.6 | 2.7 | 19.0 | 2.7 | 0.473 | -2.4 |  |
| **Insomnia** | 20.6 | 3.6 | 21.3 | 6.7 | 0.885 | -0.7 |  |
| **Pain** | 16.5 | 2.9 | 16.6 | 2.9 | 0.969 | -0.1 |  |
| **Dyspnoea** | 13.7 | 2.9 | 5.0 | 2.9 | **0.018** | 8.7 |  |
| **Financial difficulties** | 5.1 | 1.7 | 3.0 | 1.7 | 0.340 | 2.1 |  |
| **Constipation** | 6.4 | 2.4 | 9.5 | 2.4 | 0.307 | -3.1 |  |
| **Diarrhoea** | 5.7 | 2.1 | 7.9 | 2.1 | 0.379 | -2.2 |  |
| **Appetite loss** | 5.0 | 1.4 | 0.3 | 1.4 | **0.009** | 4.7 |  |
| **Nausea and vomiting** | 1.9 | 0.6 | 1.0 | 0.6 | 0.234 | 0.9 |  |
| **EORTC PR-25 scales** |  |  |  |  |  |  |  |
| **Urinary symptoms** | 15.5 | 2.1 | 16.9 | 2.1 | 0.605 | -1.4 |  |
| **Urinary bother^1^** | 18.8 | 11.0 | 25.7 | 8.3 | 0.558 | -6.9 |  |
| **Bowel symptoms** | 4.9 | 1.2 | 4.2 | 1.2 | 0.644 | 0.7 |  |
| **Hormonal treatment-related symptoms** | 10.0 | 1.5 | 10.6 | 1.5 | 0.722 | -0.6 |  |
| **Sexual activity** | 43.8 | 4.0 | 46.3 | 4.0 | 0.611 | -2.5 |  |
| **Sexual functioning^1^** | 43.0 | 4.2 | 42.9 | 3.4 | 0.982 | 0.1 |  |
| EORTC QLQ-C30: higher scores indicated better function or global health but more symptom complaints.  EORTC PR-25: higher score represents a greater symptom burden or better sexual functioning and activity.  Mean scores were adjusted for age at survey, years since diagnosis and cancer stage.  Information about NSRP status was complete.  ^1^ smaller sample size as questions regarding these functions were conditional – urinary bother (n=37) & sexual functioning (n=73) | | | | | | | |

| **Table S3: Distribution of nerve-sparing status before and after multiple imputation** | | | | |
| --- | --- | --- | --- | --- |
|  | **Before Imputation** | | | **After Imputation^1^** |
|  | **Including Missing Values** | | **Complete Case** |  |
| **Therapies** | **n** | **Col%** | **Col%** | **%** |
| Non-Nerve-Sparing Radical Prostatectomy | 88 | 23.0 | 40.0 | 43.7 |
| Nerve-Sparing Radical Prostatectomy (unilateral/bilateral) | 132  (65/67) | 34.6 | 60.0 | 56.3 |
| Missing | 162 | 42.4 | - | - |
| Total | 382 | 100.0 | 100.0 | 100.0 |
| ^1^Missing data were imputed using the MICE (Multiple Imputation Chained Equations)  procedure with 25 repetitions | | | | |

| **Table S~~4~~:** **Demographic and clinical characteristics of PC survivors by nerve-sparing status (complete case)** | | | | |
| --- | --- | --- | --- | --- |
|  | **Total** | **Non-NSRP** | **NSRP** | **Non-NSRP vs. NSRP** |
|  | (n=220) | (n=88) | (n=123) |  |
|  | **Col%** | **Col%** | **Col%** | **p-value** |
| **Age at survey** |  |  |  |  |
| <70 years | 29.1 | 29.6 | 28.8 |  |
| 70-74 years | 30.5 | 27.3 | 32.6 |  |
| 75-79 years | 25.9 | 23.9 | 27.3 |  |
| ≥80 years | 14.6 | 19.3 | 11.4 | 0.503 |
| Mean (SD) | 72.4 (6.3) | 73.2 (6.5) | 72.5 (6.2) | 0.405 |
| **Education^1^ (highest degree)** |  |  |  |  |
| Low | 0.4 | 0.0 | 0.8 |  |
| Medium | 57.3 | 52.3 | 60.6 |  |
| High | 42.3 | 47.7 | 38.6 | 0.165 |
| **Nationality Swiss (yes)** | 96.8 | 97.7 | 96.2 | 0.530 |
| **Language questionnaire** |  |  |  |  |
| German | 90.9 | 94.3 | 88.6 |  |
| French/Italian | 8.1 | 5.7 | 11.4 | 0.155 |
| **Living with partner (yes)** | 82.2 | 80.3 | 83.5 | 0.570 |
| **Working at survey (yes)** | 11.4 | 11.4 | 11.4 | 1.000 |
| **Body-Mass-Index** |  |  |  |  |
| <18.5 | 0.5 | 1.1 | 0.0 |  |
| 18.5–24.9 | 33.3 | 29.6 | 35.9 |  |
| 25.0–29.9 | 53.7 | 52.3 | 54.7 |  |
| ≥30 | 12.5 | 17.0 | 9.4 | 0.198 |
| **Cancer stage** |  |  |  |  |
| T2N0M0 | 78.2 | 71.6 | 82.6 |  |
| T3N0M0 | 21.8 | 28.4 | 17.4 | 0.054 |
| **Years since diagnosis** |  |  |  |  |
| 5-6 | 28.2 | 23.9 | 31.1 |  |
| 7-8 | 44.6 | 40.9 | 47.0 |  |
| 9-10 | 27.2 | 35.2 | 21.9 | 0.047 |
| Mean (SD) | 7.5 (1.4) | 7.8 (1.5) | 7.3 (1.5) | 0.021 |
| **Disease progression/relapse** | 21.9 | 28.7 | 17.4 | 0.048 |
| **Comorbidities at diagnosis** |  |  |  |  |
| 0 | 70.5 | 76.2 | 66.7 |  |
| 1 | 19.9 | 14.3 | 23.7 |  |
| 2 | 9.6 | 9.5 | 9.6 | 0.299 |
| **Further therapy (during 1^st^ year after diagnosis)** |  |  |  |  |
| External-beam radiation therapy | 4.1 | 4.6 | 3.8 | 0.768 |
| Hormone therapy | 1.4 | 1.2 | 1.5 | 0.827 |
| Col. – Column  ^1^ Education: Low (no or primary school); Medium (lower general secondary education or vocational training); High (pre-university education, high vocational training, university) | | | | |

| **Table S5: Adjusted mean EORTC QLQ-C30 and EORTC PR-25 scores of PC survivors by nerve-sparing status (complete case)** | | | | | | | |
| --- | --- | --- | --- | --- | --- | --- | --- |
|  | **Non-NSRP** | | **NSRP** | | **Difference Non-NSRP - NSRP** | | |
|  | **Mean** | **SE** | **Mean** | **SE** | **p-value** | **Mean** |  |
| **EORTC QLQ-C30 Functioning scales** |  |  |  |  |  |  |  |
| **Global health/QoL** | 83.2 | 2.0 | 81.6 | 1.6 | 0.670 | 1.6 |  |
| **Physical functioning** | 93.5 | 1.7 | 94.0 | 1.4 | 0.753 | -0.5 |  |
| **Cognitive functioning** | 94.1 | 2.4 | 93.3 | 1.9 | 0.636 | 0.8 |  |
| **Emotional functioning** | 86.9 | 2.1 | 89.4 | 1.7 | 0.261 | -2.5 |  |
| **Role functioning** | 87.6 | 2.4 | 90.5 | 1.9 | 0.263 | -3.0 |  |
| **Social functioning** | 89.6 | 2.9 | 88.2 | 2.3 | 0.590 | 1.4 |  |
| **EORTC QLQ-C30 Symptom scales** |  |  |  |  |  |  |  |
| **Fatigue** | 12.6 | 2.6 | 12.9 | 2.1 | 0.874 | -0.3 |  |
| **Insomnia** | 14.0 | 3.5 | 17.3 | 2.9 | 0.430 | -3.3 |  |
| **Pain** | 10.3 | 2.5 | 12.3 | 2.0 | 0.474 | -2.0 |  |
| **Dyspnoea** | 7.8 | 2.7 | 5.9 | 2.2 | 0.380 | 1.9 |  |
| **Constipation** | 6.6 | 2.0 | 5.6 | 1.6 | 0.549 | 1.0 |  |
| **Diarrhoea** | 4.5 | 1.9 | 5.8 | 1.5 | 0.564 | -1.3 |  |
| **Appetite loss** | 3.3 | 1.3 | 1.2 | 1.1 | 0.201 | 2.1 |  |
| **Nausea and vomiting** | 1.5 | 0.7 | 0.4 | 0.5 | 0.217 | 1.1 |  |
| **Financial difficulties** | 2.8 | 1.1 | 1.9 | 0.9 | 0.589 | 0.9 |  |
| **EORTC PR-25 scales** |  |  |  |  |  |  |  |
| **Urinary symptoms** | 15.0 | 2.1 | 14.6 | 1.7 | 0.759 | 0.4 |  |
| **Urinary bother^1^** | 10.9 | 7.1 | 11.1 | 5.2 | 0.872 | -0.2 |  |
| **Bowel symptoms** | 3.8 | 1.0 | 2.8 | 0.8 | 0.319 | 1.0 |  |
| **Hormonal treatment-related symptoms** | 10.9 | 1.5 | 8.4 | 1.2 | 0.118 | 2.5 |  |
| **Sexual activity** | 40.2 | 4.2 | 48.7 | 3.4 | **0.041** | -8.5 |  |
| **Sexual functioning^1^** | 44.7 | 4.0 | 43.5 | 3.1 | 0.921 | 1.2 |  |
| EORTC QLQ-C30: higher scores indicated better function or global health but more symptom complaints.  EORTC PR-25: higher score represents a greater symptom burden or better sexual functioning and activity.  Mean scores were adjusted for age at survey, years since diagnosis, cancer stage, comorbidities at diagnosis further therapy if appropriate.  ^1^ smaller sample size as questions regarding these functions were conditional – urinary bother (n=42) & sexual functioning (n=81) | | | | | | | |

| **Table S6: Adjusted mean EORTC QLQ-C30 and EORTC PR-25 scores of PC survivors aged younger than 72.4 years by nerve-sparing status (after multiple imputation of missing values)** | | | | | | | |
| --- | --- | --- | --- | --- | --- | --- | --- |
|  | **Non-NSRP** | | **NSRP** | | **Difference Non-NSRP - NSRP** | | |
|  | **Mean** | **SE** | **Mean** | **SE** | **p-value** | **Mean** |  |
| **EORTC QLQ-C30 Functioning scales** |  |  |  |  |  |  |  |
| **Global health/QoL** | 80.7 | 2.9 | 80.0 | 2.4 | 0.81 | 0.7 |  |
| **Physical functioning** | 93.5 | 1.6 | 94.0 | 1.4 | 0.714 | -0.5 |  |
| **Cognitive functioning** | 91.4 | 2.9 | 89.8 | 2.5 | 0.637 | 1.6 |  |
| **Emotional functioning** | 83.2 | 2.6 | 85.8 | 2.2 | 0.422 | -2.6 |  |
| **Role functioning** | 88.6 | 2.1 | 91.0 | 1.8 | 0.289 | -2.4 |  |
| **Social functioning** | 86.4 | 3.3 | 86.5 | 2.8 | 0.917 | -0.1 |  |
| **EORTC QLQ-C30 Symptom scales** |  |  |  |  |  |  |  |
| **Fatigue** | 16.1 | 3.0 | 15.9 | 2.5 |  | 0.2 |  |
| **Insomnia** | 18.5 | 3.7 | 18.9 | 3.2 | 0.964 | -0.4 |  |
| **Pain** | 13.5 | 2.9 | 14.2 | 2.5 | 0.974 | -0.7 |  |
| **Dyspnoea** | 11.5 | 2.6 | 8.5 | 2.1 | 0.46 | 3.0 |  |
| **Constipation** | 2.5 | 1.2 | 2.3 | 1.0 | 0.946 | 0.2 |  |
| **Diarrhoea** | 5.0 | 2.3 | 6.7 | 2.0 | 0.592 | -1.7 |  |
| **Appetite loss** | 4.3 | 1.7 | 3.3 | 1.5 | 0.664 | 1.0 |  |
| **Nausea and vomiting** | 4.0 | 1.7 | 1.6 | 1.5 | 0.286 | 2.4 |  |
| **Financial difficulties** | 3.4 | 1.2 | 1.7 | 1.0 | 0.271 | 1.7 |  |
| **EORTC PR-25 scales** |  |  |  |  |  |  |  |
| **Urinary symptoms** | 16.4 | 2.3 | 16.4 | 1.9 | 0.984 | 0.0 |  |
| **Urinary bother^1^** | 34.6 | 12.0 | 29.2 | 9.6 | 0.701 | 5.4 |  |
| **Bowel symptoms** | 5.9 | 1.3 | 5.3 | 1.1 | 0.743 | 0.6 |  |
| **Hormonal treatment-related symptoms** | 14.2 | 1.6 | 10.8 | 1.4 | 0.135 | 3.4 |  |
| **Sexual activity** | 46.1 | 4.2 | 52.4 | 3.6 | 0.289 | -6.3 |  |
| **Sexual functioning^1^** | 48.1 | 3.3 | 50.8 | 2.6 | 0.664 | -2.7 |  |
| EORTC QLQ-C30: higher scores indicated better function or global health but more symptom complaints.  EORTC PR-25: higher score represents a greater symptom burden or better sexual functioning and activity.  Mean scores were adjusted for age at survey, years since diagnosis, cancer stage, comorbidities at diagnosis further therapy if appropriate.  ^1^ smaller sample size as questions regarding these functions were conditional – urinary bother (n=30) & sexual functioning (n=105) | | | | | | | |

| **Table S7: Adjusted mean EORTC QLQ-C30 and EORTC PR-25 scores of PC survivors aged 72.4 years or older by nerve-sparing status (after multiple imputation of missing values)** | | | | | | | |
| --- | --- | --- | --- | --- | --- | --- | --- |
|  | **Non-NSRP** | | **NSRP** | | **Difference Non-NSRP - NSRP** | | |
|  | **Mean** | **SE** | **Mean** | **SE** | **p-value** | **Mean** |  |
| **EORTC QLQ-C30 Functioning scales** |  |  |  |  |  |  |  |
| **Global health/QoL** | 76.4 | 2.3 | 76.2 | 2.0 | 0.939 | 0.2 |  |
| **Physical functioning** | 87.5 | 2.2 | 88.0 | 1.9 | 0.836 | -0.5 |  |
| **Cognitive functioning** | 83.4 | 3.2 | 84.6 | 2.9 | 0.804 | -1.2 |  |
| **Emotional functioning** | 84.2 | 2.5 | 85.4 | 2.2 | 0.716 | -1.2 |  |
| **Role functioning** | 81.8 | 2.5 | 86.0 | 2.2 | 0.173 | -4.2 |  |
| **Social functioning** | 85.0 | 3.1 | 85.1 | 2.8 | 0.934 | -0.1 |  |
| **EORTC QLQ-C30 Symptom scales** |  |  |  |  |  |  |  |
| **Fatigue** | 22.2 | 2.9 | 20.2 | 2.6 | 0.48 | 2.0 |  |
| **Insomnia** | 21.7 | 3.7 | 22.0 | 3.3 | 0.968 | -0.3 |  |
| **Pain** | 16.7 | 3.0 | 19.7 | 2.8 | 0.668 | -3.0 |  |
| **Dyspnoea** | 12.0 | 3.2 | 12.2 | 3.0 | 0.948 | -0.2 |  |
| **Constipation** | 4.4 | 2.9 | 4.5 | 2.0 | 0.787 | -0.1 |  |
| **Diarrhoea** | 10.4 | 3.2 | 10.1 | 2.9 | 0.981 | 0.3 |  |
| **Appetite loss** | 7.5 | 2.5 | 7.9 | 2.3 | 0.78 | -0.4 |  |
| **Nausea and vomiting** | 6.3 | 2.0 | 5.0 | 1.9 | 0.622 | 1.3 |  |
| **Financial difficulties** | 1.6 | 0.6 | 0.8 | 0.5 | 0.352 | 0.8 |  |
| **EORTC PR-25 scales** |  |  |  |  |  |  |  |
| **Urinary symptoms** | 20.8 | 2.1 | 19.0 | 1.9 | 0.517 | 1.8 |  |
| **Urinary bother^1^** | 32.2 | 7.6 | 29.7 | 6.3 | 0.716 | 2.5 |  |
| **Bowel symptoms** | 4.9 | 1.2 | 4.6 | 1.1 | 0.89 | 0.3 |  |
| **Hormonal treatment-related symptoms** | 13.2 | 1.7 | 11.1 | 1.6 | 0.313 | 2.1 |  |
| **Sexual activity** | 32.4 | 3.5 | 42.6 | 3.1 | **0.043** | -10.2 |  |
| **Sexual functioning^1^** | 48.4 | 4.9 | 42.6 | 3.9 | 0.402 | 5.8 |  |
| EORTC QLQ-C30: higher scores indicated better function or global health but more symptom complaints.  EORTC PR-25: higher score represents a greater symptom burden or better sexual functioning and activity.  Mean scores were adjusted for age at survey, years since diagnosis, cancer stage, comorbidities at diagnosis further therapy if appropriate.  ^1^ smaller sample size as questions regarding these functions were conditional – urinary bother (n=74) & sexual functioning (n=78) | | | | | | | |
